# Nationwide Survey of Knowledge and Health Beliefs regarding Human Papillomavirus among HPV-Vaccinated Female Students in Malaysia

**DOI:** 10.1371/journal.pone.0163156

**Published:** 2016-09-22

**Authors:** Li Ping Wong, Raja Nur Amalina Raja Muhammad Yusoff, Zobaida Edib, I-Ching Sam, Gregory D. Zimet

**Affiliations:** 1 Department of Social and Preventive Medicine, Faculty of Medicine, University of Malaya, 50603 Kuala Lumpur, Malaysia; 2 Department of Medical Microbiology, Faculty of Medicine, University of Malaya 50603 Kuala Lumpur, Malaysia; 3 Department of Pediatrics, School of Medicine, Indiana University, 410 W, 10th St., HS 1001, Indianapolis, IN 46202, United States of America; Universidade Estadual de Maringa, BRAZIL

## Abstract

The National HPV Immunization Programme, which offers free human papillomavirus (HPV) vaccines to teenaged female students, was launched in Malaysia in 2010. HPV vaccination paired with adequate knowledge about HPV infection provides the best protection against cervical cancer. To identify the level of knowledge and the health beliefs towards HPV and the HPV vaccine among HPV-vaccinated female students in Malaysia. A nationwide cross-sectional survey among 14 years old female students who had received three doses of the HPV vaccine was conducted in 32 randomly selected schools from 13 states and 3 federal territories in Malaysia between February 2013 and April 2013. Among 2482 respondents, knowledge about HPV infection and the HPV vaccine was extremely poor. The mean total knowledge score was only 3.56 (SD ± 1.76), out of a possible score of 10. The majority of respondents were unaware that *vaccinating boys with HPV can help protect girls against HPV infection* (91.6%), *HPV cannot be cured* (81.6%) and that *HPV is a sexually transmitted infection* (70.3%). Most of the respondents had the misconception that *only females get HPV* (95.1%), and that *the HPV vaccine eliminates the need for Pap smear tests* (68.3%). Most respondents (91.6%) believed that they would not get an HPV infection. Almost half of the respondents (42.9%) held the misconception that HPV infection could not lead to serious illness. Findings revealed poor knowledge about both HPV and the HPV vaccine, low perceived susceptibility to HPV infection and misinformation about HPV infection among HPV-vaccinated girls. Therefore, it is essential to increase the knowledge and awareness of health risks regarding HPV infection among teenaged girls who have received the HPV vaccine.

## Introduction

Cervical cancer is one of the leading causes of death in women around the world [1]. According to the World Health Organization, cervical cancer is the fourth most common cancer in women after breast, colorectal and lung cancers and it accounts for more than 528,000 new cancer cases each year [2]. Oncogenic human papillomavirus (HPV) types 16 and 18 account for about 74% of cervical cancers as well as other cancers of the anus, penis, vulva, vagina, mouth and oropharynx [3]. Low-risk type infections (e.g. HPV types 6 and 11) are responsible for benign or low-grade cervical cell changes, genital warts and laryngeal papillomas [4].

Vaccines are now available to prevent HPV types 16 and 18 which most commonly cause cervical cancer and its precursors [5]. HPV vaccination is most effectively given during early adolescence, before a person becomes sexually active during early adolescence [6]. The Advisory Committee on Immunization Practices of the Centres for Disease Control and Prevention and the American Academy of Pediatrics recommend routine vaccination for girls aged 11 or 12 years [7]. In Malaysia, a National HPV Immunization Programme was launched by the Ministry of Health in 2010, offering Form One female students (13 years old) the bivalent HPV vaccine, given in three doses over six months with parental consent [8]. According to Annual Report of Malaysia Ministry of Health (2011), the immunisation coverage for complete 3 dosage of HPV vaccine among girls aged 13 years old has been more than 95% since it has been introduced in 2010 and accompanied by targeted public health information provided by school health teams [8]. The education component of the National HPV Immunization Program is unclear. Nevertheless, based on our inquiry prior the onset of this study, the provision of health information and its content were not consistent. In some schools, information was delivered by school teachers and not the health teams. Some schools even reported that no health information was given along with the vaccine. Although no empirical evidence that showed HPV vaccination is more effective if accompanied by improved knowledge, imparting HPV-related knowledge increases awareness regarding HPV is important [9]. We postulate that education to enhance knowledge should be carried out in addition to the provision of HPV vaccination in order for the vaccination programme to be truly effective. Studies shows that while vaccinated teenaged students have a better overall knowledge of HPV compared to unvaccinated students [10], misconceptions about the HPV and its association with cervical cancer remain [9–11].

Along with knowledge, health beliefs about HPV are also important. The Health Belief Model (HBM) is the most widely used theoretical framework to study personal beliefs that predict disease knowledge and human health behaviours, including risky sexual behaviour, intention to receive HPV vaccine, and practice of preventive measures against HPV [12, 13]. A lack of knowledge or misbelief of the potential seriousness of HPV infection may also discourage women from adopting preventive vaccine and non-vaccine measures [14]. For example, in one study, most of the young women surveyed held the misbelief that they were not susceptible to HPV infection if they were not engaging in any risky sexual practices, and this reduced their intent to receive the HPV vaccine [15]. Further, misbeliefs may prevent females from achieving full protection against cervical cancer. For example, many females may be unaware that the HPV vaccine they received does not protect them against all cancer causing HPV types, which may result in forgoing cervical cancer screening in the future. Therefore, it is essential to provide good knowledge in addition to giving the HPV vaccine. In Malaysia, the level of knowledge about HPV and the HPV vaccine of girls who have received the HPV vaccine has not been investigated. It is unknown if the information provided along with the HPV vaccination enhanced the level of knowledge among the girls who have received the HPV vaccine. Therefore, this study was carried out to explore the level of knowledge and the health beliefs regarding HPV infection and the vaccine among HPV vaccinated female students in Malaysia.

## Methods

### Sampling frame

A nationwide survey was conducted by simple random sampling to select two secondary schools from each of the 13 states and 3 federal territories in Malaysia, giving a total of 32 schools. All Form Two Malaysian female students (aged 14 years) in the sampled schools who had completed the three doses of the HPV vaccine as part of National HPV Immunization Programme in the previous year were invited to participate in the survey. The study was carried out between February 2013 and April 2013.

### Instruments

A standardized questionnaire was given to each student. The questionnaire assessed demographic characteristics, and knowledge and health beliefs related to HPV infection and vaccination. In the knowledge section, respondents were asked a series of questions regarding HPV infection, its relationship to cervical cancer and genital warts, HPV vaccine and the effectiveness of the HPV vaccine (10 item scales), and were asked to choose only one reply from three given choices of ‘true’, ‘false’ and ‘don’t know’. A correct response was given a score of one, and an incorrect or ‘don’t know’ was scored as zero. The possible total knowledge score ranged from 0 to 10, with higher scores representing higher levels of knowledge.

The second section of the questionnaire assessed respondents’ health beliefs towards HPV vaccination using the HBM construct as the theoretical framework [13], which includes general heath beliefs (2 items), perceived benefits of HPV vaccine (1 item), perceived susceptibility to HPV (1 item), worry about getting infected with HPV (1 item) and perceived severity of HPV infection (1 item). For each statement, respondents could choose only one of three response categories of ‘agree’, ‘don’t know’ and ‘disagree’.

The third section of the questionnaire assessed respondents’ attitudes towards HPV vaccination. Seven statements were presented, and respondents could choose only one from three response categories of ‘agree’, ‘don’t know’ and ‘disagree’.

The questionnaire was in three languages: Bahasa Melayu (Malay), Chinese and English, and was pretested before commencing the study. At each selected school, a survey administrator was appointed and briefed on how to correctly administer the survey questionnaires.

### Pretesting and validation of study instruments

The questionnaire was content validated by three experts from the Department of Social and Preventive Medicine of the University of Malaya to ensure the relevance (or the intent) and clarity of the questions. After minor amendments, the questionnaire was pilot tested on 20 randomly sampled students who were not included in the actual study. Pilot test participants were included from the three main ethnic groups, the Malays, Chinese and Indians. The questionnaire was revised again according to the students’ comprehension of the questions. Then, the questionnaire was construct validated on 150 randomly sampled students and factor loading was calculated to indicate the level of each specific knowledge item. The internal consistency (Cronbach’s alpha) of the score for knowledge items was found to be 0.653. After two weeks, test-retest reliability was assessed on the same 80 participants, and found to be 0.2 to 0.5, with most kappa values greater than 0.1. Based on the outcomes of the construct validity and test-retest, the questionnaire was revised again before the actual study was done.

### Ethical considerations

The study was approved by the Medical Ethics Committee at the University of Malaya Medical Centre, Kuala Lumpur, Malaysia and the Ministry of Education, Malaysia (reference: number 968.3). Written permission was obtained from the State Education Department in each of the states and federal territories. Written permission was further obtained from the principals of the selected schools. Written informed consent was also obtained from the students, and their participation in the study was voluntary.

### Statistical analysis

All statistical analyses were performed with Statistical Package for the Social Sciences version 20.0 (IBM, New York, USA). The significance level was set at *p* < 0.05. Descriptive statistics include the calculation of frequencies for demographic variables, knowledge and health belief items, and mean scores for the knowledge items. Independent t-test and one way analysis of variance (ANOVA) were used for comparison of means and to test the associations between the total knowledge score and demographic variables.

## Results

### Respondents’ characteristics

A summary of the respondent characteristics is provided in Table 1. Of the 3094 Form Two female students in the selected schools, 2482 respondents participated in the survey, giving a response rate of 80.2%. The mean age of the respondents was 14.0 years (SD ± 0.18). The majority of the respondents were Malay (63.3%), followed by Chinese (23.2%) and Indian (4.8%), which is similar to the national distribution of ethnicities. More than half of them were Muslims (65.8%), followed by Buddhists (18.2%), Christians (8.2%), Hindus (4.7%), Taoists (2.7%) and others (0.4%). A total of 77.5% reported being moderately religious, while 20.7% were very religious, and 1.8% were not religious at all. More than 75% of the respondents’ mothers were semi-professionals and/or housewives (80.3%), while the majority of their fathers’ occupations were semi-professional or unemployed (68.8%). Most of the respondents (71.4%) were from households with a monthly income below MYR 3000.

**Table 1:** Demographic Characteristics of Respondents (n = 2482).

Socio-demographic variables	Total sample	
	N	%
**Ethnicity**		
Malay	1570	63.3
Chinese	577	23.2
Indian	120	4.8
Others	215	8.7
**Religion**^**a**^		
Muslim	1629	65.8
Buddhist	452	18.2
Taoist	68	2.7
Hindu	117	4.7
Christian	203	8.2
Others	9	0.4
**Religious status**^**a**^		
Very religious	505	20.7
Moderately religious	1886	77.5
No at all religious	45	1.8
**Mother’s occupation**		
Professional or managerial	488	19.7
Semi-professional or housewife	1993	80.3
**Fathers occupation**^**a**^		
Professional or managerial	775	31.2
Semi-professional or unemployed	1705	68.8
**Monthly Household Income†**		
≤ MYR 3000	1612	71.4
> MYR 3000	648	28.6

^a^Number of respondents less than 2482 (total respondents) due to non-response.

†1 USD = 3.80 Malaysian Ringgit (MYR) as of 19 July, 2015.

### Knowledge

The respondents had received information about HPV infection or vaccination from a variety of sources, including a doctor (53.8%), a teacher (53.4%), television or radio (29.6%), parents (28%), newspapers (21.3%), friends (13.4%), and the internet (11.5%) (Table 2).

**Table 2:** Source of Information on HPV.

Information source	N^a^	Yes (%)	No (%)
Parents	2480	28.0	72.0
Teacher	2480	53.4	46.6
Peers	2480	13.4	86.6
Newspaper or magazine	2480	21.3	78.7
Television or radio	2480	29.6	70.4
Internet	2480	11.5	88.5
Doctor	2480	53.8	46.2
Others	2480	1.2	98.8

^a^Number of respondents less than 2482 (total respondents) due to non-response.

Table 3 shows that of the respondents, a majority (85.7%) agreed that *only females get HPV infection* and more than two-thirds (72.4%) did not know that *genital warts are caused by HPV*. Nearly half of the respondents disagreed with the statement that *vaccinating boys against HPV can help to protect female students against HPV infection* (42.2%). Half of the respondents did not know the following that: *the HPV vaccine does not eliminate the need for Pap smear tests* (51.2%), *HPV is a sexually transmitted infection* (47.6%), *HPV cannot be cured* (45.2%) and *HPV infections are common and many people have been infected* (43.8%). A majority correctly responded that *vaccines are available to prevent HPV infection* (79.6%), *HPV can cause cervical cancer* (71.1%) and *most people who become infected with HPV do not even know it* (66.4%).

**Table 3:** Response for knowledge items.

HPV knowledge items	True	False	Do not know
	%	%	%
HPV infections are common and many have been infected	32.4	23.8	43.8
Most people who become infected with HPV do not even know they have it	66.4	6.3	27.3
Only females get HPV infection	85.7	4.9	9.4
HPV can cause cervical cancer	71.1	7.6	21.3
Genital warts are caused by HPV	14.7	12.9	72.4
HPV is a sexually transmitted infection	29.7	22.7	47.6
HPV cannot be cured	18.4	36.4	45.2
Vaccines are available to prevent HPV infection	79.6	3.8	16.6
The HPV vaccine gets rid of the need for Pap smears tests	17.1	31.7	51.2
Vaccinating boys with HPV can help protect girls against HPV infection	8.4	42.2	49.4

Across the sample, the mean knowledge score (10 items) was only 3.56 (SD ± 1.76; 95% CI 3.49–3.63) out of a possible score of 10. As shown in Table 4, the mean total knowledge score was significantly higher among Malay students (3.68, SD ± 1.67) than among other ethnicities (*p* = 0.0001). It was also found that the mean total knowledge score was significantly higher among respondents with mothers in professional and managerial capacities (3.72; SD ± 1.64, *p* = 0.018) and likewise fathers (3.68; SD ± 1.72, *p* = 0.027) than among others. The total knowledge score was also significantly higher among respondents from households with a monthly income above MYR 3000 than from households with an income < MYR 3000 (3.71; SD ± 1.69, *p* = 0.019).

**Table 4:** Association between demographic characteristics and mean knowledge.

Characteristics	All respondents	Mean score (± SD)	
	(N^b^)		
		Total knowledge	*p* value
		(0–10 items scale)	
**Ethnicity**			
Malay	1514	3.68 (1.67)	< 0.001*
Chinese	548	3.28 (1.86)	
Indian	109	3.13 (1.90)	
Others	209	3.65 (1.89)	
**Religious status**^**a**^			
Very religious	485	3.53 (1.71)	0.672
Moderately religious	1818	3.57 (1.76)	
Not at all religious	40	3.35 (2.03)	
**Mother’s occupation**			
Professional or managerial	314	3.72 (1.64)	0.018*
Semi-professional or Housewife	159	3.51 (1.78)	
**Fathers occupation**^**a**^			
Professional or managerial	415	3.68 (1.72)	0.027*
Semi-professional or unemployed	336	3.51 (1.77)	
**Monthly Household Income**			
≤MYR3000	1612	3.53(1.75)	0.019*
> MYR3000	648	3.71 (1.69)	

^a^Respondents with missing information on a covariate were excluded from the analysis.

^b^Subtotal may vary owing to missing data.

*p < 0.05 is considered to be significant.

### Health beliefs

Table 5 shows that 72.3% of respondents believed that *the HPV vaccine is good for one’s health just like all other vaccines*, and 80.1% believed that *taking the HPV vaccine is a good idea as it is recommended by the government*. The majority of respondents (84.5%) believed that *the HPV vaccination is beneficial*. More than half (67%) were worried about getting infected with HPV and had the belief that *infection with HPV can lead to serious illness* (57.1%). Only 8.4% of respondents felt that *they were susceptible to getting infected with HPV*. Most respondents (84.2%) reported that they thought that *HPV vaccine shots will prevent HPV infec*tion.

**Table 5:** Health beliefs towards HPV vaccination.

Statements	n^a^	Agree	Disagree	Don’t know
		n (%)	n (%)	n (%)
**General health beliefs**				
HPV vaccine is good for health just like all other vaccines	2439	1764 (72.3)	83 (3.4)	592 (24.3)
Taking the HPV vaccine is a good idea because it is recommended by the government	2428	1987 (81.8)	69 (2.9)	372 (15.3)
**Perceived benefit**				
The HPV vaccine would be a good way to prevent HPV infection	2438	2059 (84.5)	47 (1.9)	332 (13.6)
**Perceived susceptibility toward HPV**				
Do you think you will get infected with HPV?	2440	206 (8.4)	536 (22.0)	1698 (69.6)
**Feeling of worry**				
I am worried about getting infected with HPV	2447	1639 (67.0)	294 (12.0)	514 (21.0)
**Perceived severity**				
Infection with HPV can lead to serious illness	2445	1395 (57.0)	141 (5.8)	909 (37.2)

^a^Number of respondents less than 2482 (total respondents) due to non-response.

### Attitudes towards HPV vaccination

Table 6 represents respondents’ attitudes towards HPV vaccination. Almost a third of respondents (27.3%) reported that fear of vaccinations and pain were barriers to receiving the vaccine. Only a few respondents agreed that *My religion prohibits me from receiving the HPV vaccine because it is sex related* (1.7%), *HPV vaccine shots may encourage people to have multiple sexual partners* (3.5%), *My parents might not allow me to get the HPV vaccine* (4.1%), and *HPV vaccines are not safe for me* (7.2%).

**Table 6:** Attitudes towards HPV vaccination.

Statements	n^a^	Agree	Disagree	Don’t know
		n (%)	n (%)	n (%) -
Getting vaccines are scary and painful	2445	668 (27.3)	1004 (41.1)	773 (31.6)
HPV vaccines are not safe for me	2433	174 (7.2)	1587 (65.2)	672 (27.6)
I don’t think HPV vaccine will prevent HPV infection	2442	386 (15.8)	1255 (51.4)	801 (32.8)
HPV vaccine may encourage people to have sex at an early age	2438	128 (5.3)	1118 (45.9)	1192 (48.8)
HPV vaccine may encourage people to have multiple sexual partners	2427	85 (3.5)	1247 (51.4)	1095 (45.1)
My parents might not allow me to get the HPV vaccine	2433	100(4.1)	1780 (73.2)	553 (22.7)
My religion prohibits me from receiving HPV vaccine because it is sex related	2433	41(1.7)	1717 (70.6)	675 (27.7)

^a^Number of respondents less than 2482 (total respondents) due to non-response.

## Discussion

The study demonstrates a profound knowledge deficit concerning HPV among HPV-vaccinated female students in Malaysia. Teenagers and women who have given informed consent to receive the HPV vaccine would be expected to have improved their knowledge about HPV [16, 17]. As has likewise been found in other studies, knowledge remained suboptimal among vaccinated students despite an extensive information campaign [10, 18–21]. It was reported that the knowledge deficit regarding HPV infection and the HPV vaccine may reflect a failure to impart an effective message to the target group, thus leading to poor awareness, misconceptions and confusion regarding HPV infection and vaccination and the continued need for regular HPV screening [16, 17, 22–24]. Broad educational and nationwide campaigns along with HPV vaccination programmes do seem to have a large impact on knowledge [16]. The provision of limited knowledge to students may lead to an inadequate understanding about HPV infection [20]. School-based awareness and educational programmes covering HPV infection, vaccination, disease transmission and preventive measures against it (including the continued need for regular screening and use of condoms for all sexually-transmitted infections) should be included alongside HPV vaccination to improve their understanding of HPV infection [10, 18]. Education should also be ongoing after vaccination to aid in the retention of knowledge.

Our study revealed that that the students had low perceived susceptibility to HPV. As most of the respondents were unaware that *HPV is a common sexually transmitted infection*, this may lead to a low perceived susceptibility to HPV infection. Correcting such a fallacious perception is crucial as it may lead to a greater inclination to engage in protective behaviour connected to their low perceived susceptibility to HPV infection. Therefore, health promotion programmes should emphasize that HPV vaccination reduces, but does not eliminate susceptibility to HPV infections [25]. Further, most respondents had the misconception that *only females get HPV* and did not know that *vaccinating boys can help to protect girls*. Educational campaigns at the school level will help people to realize that both genders are equally susceptible to HPV infection [25].

Many respondents held the misbelief that *the HPV vaccine will prevent all HPV infection*, and did not know that *HPV vaccine does not remove the need for Pap smears*. This indicates poor awareness that the vaccine does not protect against all HPV types that are associated with cervical cancer [26, 27] and underlines the importance of combining vaccination and screening to provide the best protection [28]. However, an important related issue in Malaysia is the poor awareness about and uptake of cervical cancer screening [29]. Educational programmes on HPV vaccination therefore provide information that cervical screening is essential after receiving the HPV vaccine.

Only half of the respondents reported hearing about HPV from teacher, may perhaps imply that targeted public health information provided by school health teams along with the vaccine did not reach all the girls that have received the HPV vaccine. Findings are in concordance with our inquiries prior to the study where some schools reported that HPV information was not provided to the students. Our findings suggest that the health information about HPV should be made compulsory along with the provision of the HPV vaccine. Over half reported receiving HPV information from doctors may imply provision of information by HPV recommending physician during their encounter with HPV recommending physicians [30]. Less than a third of students had heard about HPV from their parents, thus indicating that either the parents know very little about HPV or there is a reluctance to discuss these issues in what is a conservative society. Low levels of parental knowledge also indicate less concern about sexual behaviour at early age [31]. Nearly a third of students had heard about HPV from television, radio or newspapers, demonstrating that these traditional forms of media are less important in these target groups of teenage students. A lack of media publicity was also reported in a previous study in Malaysia [32]. National educational campaigns are required through multiple channels, including programmes at the school level, media publicity and also healthcare providers’ initiatives [33, 34].

The key limitation of this study is that all measures were based on the participants’ self-reports, which may cause reporting bias. Despite this limitation, the uniqueness of this study is that it includes a large sample from all parts of Malaysia, allowing for a reliable assessment of HPV knowledge and beliefs among the key population of HPV-vaccinated female students.

## Conclusion

Despite the provision of a free National HPV Vaccination Programme since 2010, poor knowledge and misconceptions regarding HPV infection and vaccination still exist, along with low perceived susceptibility to HPV among vaccinated teenaged female students in Malaysia. More effective education programmes are much needed to enhance knowledge and heath beliefs, and to ensure sustainable reductions of HPV infection and associated diseases.

## Supporting Information

S1 File Questionnaire. (DOCX)

S2 File SPSS Data. (SAV)
