# Supplementary material for: Nationwide Survey of Knowledge and Health Beliefs regarding Human Papillomavirus among HPV-Vaccinated Female Students in Malaysia
Source: PLoS One. 2016 Sep 22;11(9):e0163156. doi: 10.1371/journal.pone.0163156 (PMC5033318; doi:10.1371/journal.pone.0163156)
Supplement: S1 File — (DOCX) [file pone.0163156.s001.docx]

SULIT/ *CONFIDENTIAL ID # YW*

ID


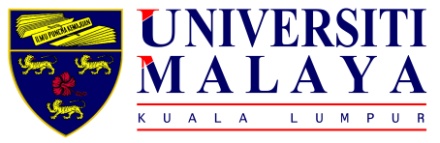


**PELAJAR PEREMPUAN TINGKATAN 2/FORM 2 GIRLS**

**Pengetahuan Hpv Dan Penerimaan Suntikan Vaksin Hpv Di Kalangan Pelajar Tingkatan 2 Perempuan /**

**Hpv Knowledge And Hpv Vaccine Acceptibility Among Form 2 Female Students/**

**中学二年级女生对HPV的认知和HPV疫苗接受程度**

# Seksyen A / Section A/A部分

# MAKLUMAT AM /GENERAL INFORMATION/基本信息

| A.1 | Nama / Name/姓名: | _____________________________________ |
| --- | --- | --- |
| A.2 | Nama Kelas / Class name/班级名: | ______________________________________ |
| A.3 | Umur pada tahun 2013 / Age in the year of 2013/2013年年龄: | ____________ Tahun / Years old/岁 |
| A.4 | Bangsa / Ethnicity/种族 | [ ] Melayu / Malay/马来族  [ ] Cina/ Chinese/华人  [ ] India/ Indian/印度人  [ ] Lain-lain, sila nyatakan/ Others, please specify/其他，请列出 :  ____________________________________ |
| A.5 | Agama / Religion/宗教 | [ ] Islam / Muslim/回教  [ ] Buddha / Buddhist/佛教  [ ] Taoist / Taoist/道教  [ ] Hindu / Hindu/兴都教  [ ] Kristian / Christian/基督教  [ ] Lain-lain, sila nyatakan/ Others, please specify/其他，请列出 :  __________________________________________ |
| A.6 | Status agama/ Religious status/宗教虔诚度 | [ ] Sangat kuat beragama / Very religious/非常虔诚  [ ] Sederhana kuat beragama / Moderately religious/虔诚  [ ] Tidak kuat beragama / Not at all religious/不虔诚 |
| A.7 | Pekerjaan Ibu / Mother’s occupation/母亲职业  Sila nyatakan/ Please indicate请指出 :  __________________________  Sila tanda kategori pekerjaan/ Please tick the occupational category/请勾出职业种类  [ ] Profesional / Professional/专业人士  [ ] Pengurusan / Managerial/经理  [ ] Semi-profesional / Semi-professional/半专业人士  [ ] Pekerja kemahiran / Skilled worker/熟练工人  [ ] Suri rumah/ Housewife/家庭主妇  [ ] Tidak berkenaan/Not applicable/不适用 | Pekerjaan Bapa / Father’s occupation/父亲职业  Sila nyatakan/ Please indicate/请指出:  __________________________  Sila tanda kategori pekerjaan/ Please tick the occupational category/请勾出职业种类  [ ] Profesional / Professional/专业人士  [ ] Pengurusan / Managerial/经理  [ ] Semi-profesional / Semi-professional/半专业人士  [ ] Pekerja kemahiran / Skilled worker/熟练工人  [ ] Tidak bekerja/ Unemployed/失业  [ ] Tidak berkenaan/Not applicable/不适用 |
| A.8 | Purata pendapatan sekeluarga sebulan / Monthly average household income/家庭平均月收入 | [ ] Di bawah RM1000 / Below RM1000/RM1000以下  [ ] RM1000 sehingga di bawah RM2000 / RM1000 – RM1999  [ ] RM2000 sehingga di bawah RM3000 / RM2000 – RM2999  [ ] RM3000 sehingga di bawah RM4000 / RM3000—RM3999  [ ] RM4000 sehingga di bawah RM5000 / RM4000—RM4999  [ ] Melebihi RM5000 / Above RM5000/RM5000以上 |

# Seksyen B/Section B/B部分

# PENGETAHUAN/KNOWLEDGE/知识

| B.1 | Sehingga sekarang, sumber maklumat HPV atau suntikan vaksin HPV yang pernah diterima adalah dari mana?/ As of today, source of HPV or HPV vaccine information you ever received?/至今你曾经获得的有关HPV和HPV疫苗的资讯来源？ | Tandakan yang mana berkenaan, boleh tanda lebih dari satu / Tick whichever applicable, can tick more than one  [ ] Ibu bapa/ Parents/父母  [ ] Cikgu/ Teacher/教师  [ ] Kawan-kawan/ Peers/同学  [ ] Surat khabar atau majalah/ Newspaper or magazine/报纸或杂志  [ ] Televisyen atau radio/ Television or radio/电视或电台  [ ] Internet/ Internet/网络  [ ] Doktor/ Doctor/  [ ] Lain-lain, sila nyatakan/ Others, please specify/其他，请指出:  ______________________________________________ |
| --- | --- | --- |

B.2 Berikut adalah soalan menguji pengetahuan tentang HPV, sila jawab “Betul”, “Salah” atau “Tidak tahu”/The following are questions about HPV, please answer “True”, “False” or “Don’t know”/下列是有关HPV的问题，请回答“是”、“不是”、或“不知道”。

| Kenyataan/Statements/陈述 | | Betul/ True/是 | Salah/ False/不是 | Tidak tahu/ Don’t know/不知道 |
| --- | --- | --- | --- | --- |
| B.2.1 | Jangkitan HPV adalah biasa dan ramai orang telah dijangkiti HPV / HPV infections are common, and many have been infected/ HPV感染是常见的，许多人已被感染。 | [ ] | [ ] | [ ] |
| B.2.2 | Kebanyakan orang yang dijangkiti HPV tidak tahu mereka telah dijangkiti/ Most people who become infected with HPV do not even know they have it/被感染的人不一定知道已被感染。 | [ ] | [ ] | [ ] |
| B.2.3 | Hanya wanita yang mendapat HPV / Only female get HPV/只有女生才会感染HPV。 | [ ] | [ ] | [ ] |
| B.2.4 | HPV biasanya tidak menyebabkan masalah kesihatan/ In most cases, HPV does not cause any health problems/通常情况HPV不会引起健康问题。 | [ ] | [ ] | [ ] |
| B.2.5 | HPV boleh menyebabkan kanser pangkal rahim (kanser serviks)/ [HPV can cause cervical cancer.](http://cpcp.sph.sc.edu/HPV/hpv.htm#cervical_cancer)/HPV会引起子宫颈癌。 | [ ] | [ ] | [ ] |
| B.2.6 | Ketuat kemaluan adalah disebabkan oleh HPV / Genital warts are caused by HPV/生殖器疣是由HPV引起的。 | [ ] | [ ] | [ ] |
| B.2.7 | HPV ialah jangkitan melalui hubungan seks / HPV is a sexually transmitted infection/HPV 是一种性传播感染 | [ ] | [ ] | [ ] |
| B.2.8 | HPV tidak boleh disembuhkan / HPV cannot be cured/HPV 是不能治愈的 | [ ] | [ ] | [ ] |
| B.2.9 | Suntikan vaksin HPV boleh didapati bagi mencegah jangkitan HPV / Vaccines are available to prevent HPV infection/疫苗可以预防HPV病毒感染。 | [ ] | [ ] | [ ] |
| B.2.10 | Dengan mengambil suntikan vaksin HPV, ujian saringan bagi mengesan kanser pangkal rahim (kanser serviks) tidak perlu dilakukan / [The HPV vaccine gets rid of the need for Pap smear tests.](http://cpcp.sph.sc.edu/HPV/hpv.htm#vaccine_pap)/ 已接种HPV疫苗就不需要再做巴氏测试。 | [ ] | [ ] | [ ] |
| B.2.11 | Lelaki yang menerima suntikan vaksin HPV boleh membantu melindungi perempuan daripada jangkitan HPV / Vaccinating boys with HPV can help protect girls against HPV infection/接种HPV疫苗的男生有助于保护女生免受HPV感染。 | [ ] | [ ] | [ ] |

# Seksyen C / Section C/C部分

# KEPERCAYAAN KESIHATAN TERHADAP SUNTIKAN VAKSIN HPV / HEALTH BELIEFS TOWARD HPV VACCINATION/与HPV疫苗接种相关的健康理念

C. Berikut adalah kenyataan tentang kepercayaan kesihatan terhadap suntikan HPV. / Following are statements regarding your health beliefs toward HPV vaccination./下列是有关疫苗接种的健康理念的陈述

| C.1 Kepercayaan kesihatan/ General health beliefs/基本健康理念 | | |
| --- | --- | --- |
| C.1.1 | Suntikan vaksin HPV adalah baik untuk kesihatan seperti suntikan yang lain/ HPV vaccine shots is good for health just like all other vaccine shots/HPV疫苗注射是如同其他疫苗一样有利于健康的接种。 | [ ] Setuju / Agree/是  [ ] Tidak tahu / Don’t know/不知道  [ ] Tidak setuju / Disagree/不认同 |
| C.1.2 | Mengambil suntikan vaksin HPV adalah baik kerana ia disarankan oleh kerajaan/ Taking the HPV vaccine shots is a good idea because it is recommended by the government/注射HPV疫苗是一个好的想法，因为这是政府倡导的。 | [ ] Setuju / Agree/是  [ ] Tidak tahu / Don’t know/不知道  [ ] Tidak setuju / Disagree/不认同 |
| C.2 Persepsi faedah / Perceived benefit/好处的认识 | | |
| C.2.1 | Suntikan vaksin HPV adalah langkah yang baik untuk mencegah jangkitan HPV / The HPV vaccine shots would be a good way to prevent HPV infection/接种HPV疫苗是一种防止HPV病毒感染的好途径。 | [ ] Setuju / Agree/是  [ ] Tidak tahu / Don’t know/不知道  [ ] Tidak setuju / Disagree/不认同 |
| C.3 Persepsi kemungkinan terhadap jangkitan HPV / Perceived susceptibility toward HPV infection/感染程度的认识 | | |
| C.3.1 | Adakah anda fikir anda mungkin mendapat jangkitan HPV? / Do you think you will get infected with HPV? /你认为你会感染HPV病毒吗？ | [ ] Setuju / Agree/是  [ ] Tidak tahu / Don’t know/不知道  [ ] Tidak setuju / Disagree/不会 |
| C.4 Perasaan kebimbangan / Feeling of worry/担忧 | | |
| C.4.1 | Saya bimbang dijangkiti HPV/ I am worry about getting infected with HPV/我担忧我会感染HPV病毒。 | [ ] Setuju / Agree/是  [ ] Tidak tahu / Don’t know/不知道  [ ] Tidak setuju / Disagree/不担忧 |
| C.5 Persepsi keterukan / Perceived severity/严重性的认识 | | |
| C.5.1 | Jangkitan HPV boleh menyebabkan penyakit yang serius / Infection with HPV can lead to serious illness/感染HPV病毒会诱发严重的病。 | [ ] Setuju / Agree/是  [ ] Tidak tahu / Don’t know/不知道  [ ] Tidak setuju / Disagree/不认同 |
| C.6 Persepsi halangan / Perceived barriers/认知上的阻碍 | | |
| C.6.1 | Suntikan vaksin adalah menakutkan dan menyakitkan/ Getting vaccine shots are scary and painful/接种疫苗是可怕和疼痛的。 | [ ] Setuju / Agree/是  [ ] Tidak tahu / Don’t know/不知道  [ ] Tidak setuju / Disagree/不是 |
| C.6.2 | Suntikan vaksin HPV tidak selamat untuk saya / HPV vaccine shots is not safe for me/HPV接种对我是不安全的。 | [ ] Setuju / Agree/是  [ ] Tidak tahu / Don’t know/不知道  [ ] Tidak setuju / Disagree/不是 |
| C.6.3 | Saya rasa suntikan vaksin HPV tidak akan mencegah jangkitan HPV / I don’t think the HPV vaccine shots will prevent HPV infection/不认为HPV疫苗注射可以预防HPV病毒感染。 | [ ] Setuju / Agree/是  [ ] Tidak tahu / Don’t know/不知道  [ ] Tidak setuju / Disagree/不是 |
| C.6.4 | Suntikan vaksin HPV boleh menggalakkan hubungan seks pada umur yang muda / HPV vaccine shots may encourage people to have sex at an early age/HPV疫苗接种会鼓励过早年龄有性行为。 | [ ] Setuju / Agree/是  [ ] Tidak tahu / Don’t know/不知道  [ ] Tidak setuju / Disagree/不是 |
| C.6.5 | Suntikan vaksin HPV boleh menggalakkan orang mempunyai ramai pasangan seks / HPV vaccine shots may encourage people to have multiple sexual partners/HPV疫苗接种在鼓励人们有更多的性伴侣。 | [ ] Setuju / Agree/是  [ ] Tidak tahu / Don’t know/不知道  [ ] Tidak setuju / Disagree/不是 |
| C.6.6 | Ibu bapa saya mungkin tidak bersetuju saya mendapatkan suntikan vaksin HPV/ My parents might not allow me to get the HPV vaccine shots/我的父母可能不允许我接种HPV疫苗。 | [ ] Setuju / Agree/是  [ ] Tidak tahu / Don’t know/不知道  [ ] Tidak setuju / Disagree/不是 |
| C.6.7 | Agama saya menghalang saya menerima suntikan vaksin HPV kerana ia berkaitan seks / My religion prohibits me from receiving HPV vaccine shots because it is sexual related/我的宗教禁止我接受HPV疫苗，因为这与性有关。 | [ ] Setuju / Agree/是  [ ] Tidak tahu / Don’t know/不知道  [ ] Tidak setuju / Disagree/不认同 |

# Seksyen D / Section D/D部分

# PENGAMBILAN SUNTIKAN VAKSIN HPV / HPV VACCINE UPTAKE/接种HPV疫苗的认识

D. Berikut adalah kenyataan tentang keinginan untuk menerima suntikan HPV/ The following are questions regarding intention to be vaccinated against HPV /下列问题是有关有意接受HPV疫苗接种的

| D.1 | Adakah anda telah menerima suntikan vaksin HPV dalam Program Pelalian HPV Kebangsaan? / Did you receive the HPV vaccine in the National HPV Immunization Program? /你有接受过国家HPV免疫项目吗？ | [ ] YA, sudah terima 3 dos lengkap / YES, received complete 3 doses/是的，整套3剂量。  **Tidak perlu jawab soalan yang seterusnya/ End of questions/结束问答** |
| --- | --- | --- |
|  |  | [ ] YA, tetapi tidak lengkap 3 dos / YES, but did not complete 3 doses./是的,但没有完成3剂量。  **Jawab D.2.1 & D.2.2 / Answer D.2.1 & D.2.2 /回答D.2.1和D.2.2** |
|  |  | [ ] Tidak menerima sebarang dos dari Program Pelalian HPV Kebangsaan / No, did not receive any dose in the National HPV Immunization Program /没有接受过国家免疫项目。  **Jawab D.3.1 & D.3.2 / Answer D.3.1 & D.3.2 /回答D.3.1和D.3.2** |
|  |  | [ ] Tidak menerima sebarang dos dari Program Pelalian HPV Kebangsaan tetapi telah menerima suntikan di hospital atau klinik. / No, did not receive any dose in the National HPV Immunization Program, but already received vaccine from hospital or clinic/没有接受过任何的国家项目，但有从医院或诊所接种疫苗。  **Tidak perlu jawab soalan yang seterusnya/ End of questions/结束问答** |
| D.2 | D. 2.1. Sebab-sebab tidak melengkapkan 3 dos / Reasons did not complete the 3 doses/没有完成3个剂量的原因 | Tandakan yang mana berkenaan, boleh tandakan lebih dari satu / Tick whichever applicable, can tick more than one./请打勾合适的答案，可多项选择  [ ] Saya terlepas peluang (tidak hadir ke sekolah semasa suntikan diberi) / I missed the opportunity (did not attend school at the time the vaccine given)/错过了时机（接种的时候不在学校）  [ ] Takut jarum/ Fear of needles/害怕打针  [ ] Kesan sampingan selepas dos pertama atau kedua / Side effect after first or second dose/第一或第二针后出现副作用。  [ ] Lain-lain, sila nyatakan / Others, please specify/其他，请指出: _________________________________________________  _________________________________________________  _________________________________________________ |
|  | D.2.2 Adakah anda berhasrat untuk menyempurnakan 3 dos tersebut pada masa hadapan? / Do you intend to complete the 3 doses in future?/你打算在将来完成3剂量吗？ | [ ] Ya / Yes/是 [ ] Tidak / No/不 |
| D.3 | D.3.1 Jika anda langsung tidak menerima suntikan vaksin HPV, sebab anda tidak menerima / If you did not receive the HPV vaccine, reasons you did not receive/如果没有接种，原因是什么？ | Tandakan yang mana berkenaan, boleh tandakan lebih dari satu / Tick whichever applicable, can tick more than one/勾出合适的答案，可以多项选择  [ ] Saya terlepas peluang (tidak hadir ke sekolah semasa suntikan diberi) / I missed the opportunity (did not attend school at the time the vaccine given)/错过了时机（接种的时候不在学校）  [ ] Tidak mempercayai suntikan vaksin sekolah, lebih memilih suntikan di hospital/klinik / Distrust school vaccination, prefer to receive vaccine in hospital/clinic/不相信学校的疫苗，乐意接受医院/诊所的疫苗  [ ] Saya terlalu muda untuk menerima suntikan tersebut / I am too young to receive the vaccine/我太小了，不适合接种  [ ] Takut jarum/ Fear of needles/害怕打针  [ ] Takut akan kesan sampingan / Afraid of side effects/担心副作用  [ ] Saya rasa suntikan HPV tidak berkesan / I don’t think HPV vaccine will work/不认为疫苗有作用  [ ] Ibu bapa tidak membenarkan kerana / Parent did not give consent because /父母不同意，因为_______________________________________________  _______________________________________________  [ ] Lain-lain, sila nyatakan / Others, please specify/其他，请指出: __________________________________________  _________________________________________________  _________________________________________________ |
|  | D.3.2 Adakah anda berhasrat untuk mengambil suntikan vaksin HPV pada masa hadapan? / Do you intend to take the HPV vaccine in future?/将来你会打算接种吗？ | [ ] Ya / Yes/会 [ ] Tidak / No/不会 |

# Seksyen E / Section E/E部分

# PENGAMBILAN KEPUTUSAN / DECISION MAKING/判断

E. Bahagian ini menilai faktor yang mempengaruhi pengambilan suntikan vaksin HPV. /This section assesses factor influence HPV vaccination decision making./这部分是评估接受HPV疫苗接种决定的影响因素

| E.1 | Faktor utama yang mempengaruhi pengambilan suntikan vaksin HPV. Tanda **SATU** jawapan sahaja/ The main factor that influence the practice of HPV vaccine uptake. Please tick only **ONE**./影响决定接种HPV疫苗的主要因素。单一选择。  [ ] Saya sendiri hendak mendapatkan suntikan HPV/ I personally decide to take the HPV vaccine/我自身决定接种HPV疫苗。  [ ] Nasihat dari guru/ Advice from teachers/ 来自教师的教导。  [ ] Ibu atau bapa memutuskan saya menerima suntikan HPV/ My parent(s) decide I should take the HPV vaccine/父母决定我要接种HPV疫苗。  [ ] Kebanyakan rakan-rakan saya telah mengambil suntikan HPV/ Many of my friends have taken the HPV vaccine /我的许多朋友有接种疫苗  [ ] Lain-lain, sila nyatakan/ Others, please specify/其他，请列出:____________________________________  _______________________________________________________________________________________ |
| --- | --- |

TERIMA KASIH / THANK YOU/谢谢
